# Perceived Resilience, Meaningful Work, and Mental Health Strain Among Emergency Medicine Clinicians Following a Surge in COVID-19

**DOI:** 10.3390/bs16010010

**Published:** 2025-12-20

**Authors:** Emma C. Vosika, Thomas W. Britt, Riley L. McCallus, Marissa Shuffler, Emily Hirsh

**Affiliations:** 1 Department of Psychology, Clemson University, Clemson, SC 29634, USAmshuffl@clemson.edu (M.S.); 2 Department of Psychology, Furman University, Greenville, SC 29613, USA; riley.mccallus1@furman.edu; 3 Department of Emergency Medicine, Prisma Health, University of South Carolina School of Medicine Greenville, Greenville, SC 29605, USA; emily.hirsh@prismahealth.org

**Keywords:** resilience, meaningful work, mental health, well-being, COVID-19

## Abstract

Emergency medicine clinicians face disproportionately high levels of burnout and mental health strain compared to other specialties. This study examined whether perceived resilience predicted reduced mental health strain following the COVID-19 Omicron surge and whether meaningful work mediated this relationship. Participants were 197 emergency medicine professionals at a large hospital system who completed monthly surveys during the pandemic. Perceived resilience and meaningful work were measured pre-Omicron surge, and mental health strain was measured post-surge. Results showed that higher perceived resilience significantly predicted lower mental health strain and that meaningful work explained 40% of this relationship. The findings emphasize that resilience matters and that its benefits are at least partly a function of meaningful work. Broader implications extend to organizations seeking to strengthen workforce well-being. Interventions should integrate resilience-building with practices that enhance purpose and meaning at work.

## 1. Introduction

Healthcare systems face unprecedented stress and immense strain during crises such as the COVID-19 pandemic, a challenge intensified by multiple surges and the emergence of variants (e.g., Delta, Omicron). These variants, each with distinct transmission rates and severity, have compounded the workload and emotional toll on healthcare professionals (Liu et al., 2022; Shi et al., 2025; Yong, 2022). Increasingly, studies have shown that healthcare workers have been among the most impacted by the COVID-19 pandemic and are at high risk for experiencing post-traumatic stress and other adverse mental health symptoms (Saladino et al., 2022; Sun et al., 2021). This is especially true for Emergency Medicine (EM) clinicians, who already work in high-stakes and unpredictable environments (Ali et al., 2017). EM personnel are often ranked among the highest medical professions to report burnout and depression (Jachmann et al., 2025; McKenna, 2024).

Past research on healthcare workforce well-being has recognized the risks of burnout in acute care settings (Chuang et al., 2016; van Mol et al., 2015). The COVID-19 pandemic introduced a new area of adversity for modern healthcare systems. The combination of sustained exposure to trauma, personal risk of infection, ethical dilemmas (e.g., resource rationing), and disruptions to employees’ recovery routines and coping mechanisms (e.g., sleep, time off, social support) created an unprecedented context. Recent reviews of this literature have examined the mental health consequences of COVID-19 (Preti et al., 2020; Saladino et al., 2020, 2022). Preti et al. (2020) highlighted the negative impact of the COVID-19 pandemic on healthcare workers’ general psychiatric, depressive, and anxiety symptoms. Saladino et al. (2020) emphasized the social impact of the COVID-19 pandemic, such as prolonged social isolation and fear of contagion of family members, which contributed to symptoms of acute psychological stress and post-traumatic stress. Building on these findings, Saladino et al. (2022) documented the high incidence of post-traumatic stress disorder in healthcare professionals during the COVID-19 pandemic.

In light of these findings, researchers and practitioners alike have emphasized the urgent need to better understand and support EM clinicians’ well-being. As such, the present study aims to investigate protective factors (i.e., perceived resilience and meaningful work) against mental health strain for healthcare workers during the heightened demands of the COVID-19 Omicron surge, which emerged in late 2021 and peaked in early 2022. By examining participants’ perceptions of their resilience before the Omicron surge and analyzing their mental health strain in the aftermath of the surge, this research explores how resilience helps healthcare professionals adapt to the unpredictable and high-pressure environment of the healthcare system during periods of extreme workload

Prior research in this area has been largely cross-sectional (e.g., AlQarni et al., 2023; Foster et al., 2024; Munn et al., 2021), leaving a critical gap in understanding how personal resources interact over time to influence mental health outcomes. Specifically, there is limited longitudinal evidence examining whether resilience not only directly protects against mental health strain but also operates indirectly through mechanisms such as meaningful work. Prior work has also been critiqued for studying resilience outside of clear adversity contexts. Britt et al. (2016) noted that resilience research often fails to account for whether individuals are functioning under conditions that constitute significant adversity.

The current study addresses these gaps by using a longitudinal design to test the relationships between perceived resilience, meaningful work, and mental health strain during a period characterized by objectively high levels of occupational adversity. EM clinicians were working in conditions characterized by sustained overcrowding, elevated patient volumes, and ongoing resource strain. All of which provide a context of adversity in which resilience processes can be meaningfully examined. With this context established, the literature offers important insights into the role of perceived resilience and meaningful work in supporting clinician well-being.

### Literature Review

Perceived resilience has emerged as a crucial protective factor that may enable healthcare workers to maintain their well-being and continue delivering their essential services (Curtin et al., 2022). A fundamental attribute important for both individual and systemic functioning in healthcare, resilience has been defined as the capacity to adapt to challenges and changes at the system level and thrive in the face of adversity (Wiig et al., 2020). Healthcare workers’ reports of resilience have been shown to predict lower mental health symptoms, including anxiety, depression, and burnout, which are often exacerbated by long work hours, isolation, and fear of infection (Yıldırım et al., 2022). Additionally, perceived resilience has been shown to help workers cope with stressors by enhancing positive emotions and supporting restorative processes such as adequate sleep (Bozdağ & Ergün, 2021; Gloria & Steinhardt, 2016; Killgore et al., 2021). In line with the well-established benefits of perceived resilience on mental health strain, we provide the following hypothesis:

**Hypothesis** **1.** *Higher perceptions of pre-Omicron surge resilience will predict lower levels of mental health strain post-Omicron surge*.

Although the benefits of perceived resilience on mental health strain are clear, the process through which perceived resilience is linked to mental health outcomes remains relatively underexplored in healthcare populations and especially EM clinicians. In a study of military personnel, Britt et al. (2021) found support for perceived resilience predicting social connection, which then predicted PTSD under high combat conditions. Within the context of healthcare, one promising pathway linking perceived resilience to lower mental health strain involves the perceived meaningfulness of work, which has been defined as the subjective experience that one’s work is purposeful, significant, and aligned with one’s values (Steger et al., 2012). Recent findings indicate that resilience is positively related to perceptions of meaningful work, suggesting that resilient individuals are more likely to view their jobs as purposeful and significant (Zheng et al., 2022). This relationship highlights that resilience may not only protect against strain directly but also shape the way employees interpret and derive value from their work.

Resilient individuals may also cognitively reframe adversity as an opportunity to learn, grow, and strengthen their sense of meaning at work (Morales-Solis et al., 2022; Tugade & Fredrickson, 2004). Rather than perceiving stressors as threatening, resilient individuals may be more likely to interpret stressors as manageable challenges that foster competence, autonomy, and connection with others (Ryan & Deci, 2000). In further support of this notion, early evidence by Smith et al. (2008) found that higher perceived resilience was significantly related to more frequent use of positive reframing coping behaviors. This cognitive reframing not only reduces the negative impact of stressors but may enable resilient employees to find value and purpose in their work through problem-solving, relationship building, and sustained engagement (Morales-Solis et al., 2022). In line with broaden-and-build theory (i.e., positive emotions expand thinking and help build long-term personal resources; Fredrickson, 2001), resilience should promote positive emotions that expand attention and cognitive flexibility, which enables individuals to see connections between their efforts and broader organizational or societal contributions (Morales-Solis et al., 2022; Tugade & Fredrickson, 2004). Together, these appraisal and resource-expansion processes allow resilient employees to extract meaning from difficult experiences and align their work with core personal values. Ultimately, resilience supports a meaning-making process that transforms adversity into purpose, which then enhances the protective effects on mental health strain. Following this rationale, we provide the following hypotheses:

**Hypothesis** **2.** *Higher perceptions of pre-Omicron surge meaningful work will predict lower levels of mental health strain post-Omicron surge.*

**Hypothesis** **3.** *Pre-Omicron surge perceptions of meaningful work will mediate the relationship between pre-Omicron surge perceived resilience and post-Omicron surge mental health strain.*

**Figure 1:**
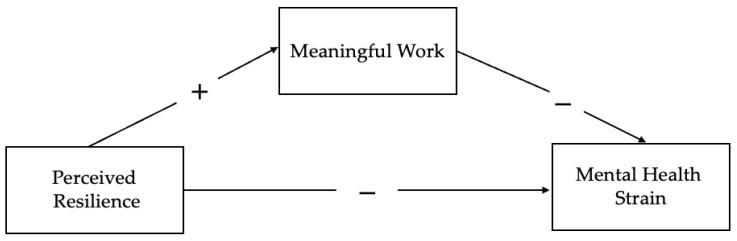
Proposed mediation model.

Figure 1 illustrates the proposed mediation model, which outlines the expected relationships between perceived resilience, meaningful work, and mental health strain. In summary, the present study examines the importance of perceived resilience and meaningful work for the mental health strain of EM clinicians working in the high-stress environment of a surge during the COVID-19 pandemic.

## 2. Materials and Methods

### 2.1. Participants and Procedure

Data were collected from a sample of healthcare professionals (*n* = 197) within the Department of Emergency Medicine (DEM) at an academic medical center in the southeastern United States. The sample was comprised of 47.7% registered nurses, 36% attending physicians, 9.1% advanced practice clinicians (APCs), and 7.1% residents. Table 1 includes further information on the sample characteristics. Participants were recruited as part of a larger quality improvement effort aimed at addressing well-being and burnout within the DEM, which included monthly surveys throughout the COVID-19 pandemic.

Perceived resilience and meaningful work were assessed in the months preceding the COVID-19 Omicron surge (November and December 2021, and January 2022). Participants were administered the Brief Resilience Scale in one of these three months. The Omicron surge reached its peak during January and February 2022 (Centers for Disease Control and Prevention, 2024). Mental health strain was assessed in February, March, and/or April 2022. Participants’ monthly post-surge responses were averaged, and within-person data were matched from the pre-surge to post-surge time periods. There was a total of 265 participants who responded to the pre-surge assessment and 241 participants who responded to the post-surge assessment. Of these participants, 197 were matched based on an anonymized ID variable.

### 2.2. Measures

Perceived resilience was measured with the Brief Resilience Scale (BRS; Smith et al., 2008), which is designed to assess the ability to recover from stress and adversity (e.g., “I tend to bounce back quickly after hard times”). The BRS consists of six items rated on a 5-point Likert scale ranging from 1 (strongly disagree) to 5 (strongly agree). Scores were averaged across items. Higher scores indicate greater perceived resilience. The BRS was utilized as it is considered one of the most valid and reliable psychometric tools for measuring perceived personal resilience (Windle et al., 2011). In the present study, the internal consistency reliability was 0.87 for the BRS.

Meaningful work was assessed using a 5-item scale designed for this survey that was developed to capture key aspects of meaningful work in healthcare settings (e.g., personal significance, patient impact, and relatedness to colleagues). Items were selected by the research team to reflect constructs found in existing measures of meaningful work (e.g., Comprehensive Meaningful Work Scale; Lips-Wiersma & Wright, 2012; Work and Meaning Inventory; Steger et al., 2012). and refined with input from EM clinicians to ensure the items were relevant and applicable to their work context. Exploratory factor analysis using principal component analysis indicated that the five items of the meaningful work scale loaded on a single factor, and the overall internal consistency reliability was 0.86. Participants were asked, “How often have the following been true for you in the past month?” and responded to the items on a 5-point scale ranging from 1 (Never/Hardly Ever) to 5 (Always). Example items include “I felt that my work was meaningful,” “I positively impacted patients and their families,” and “I left work in a positive mood.”

To assess mental health strain, we used an adapted version of the Physician Well-Being Index (PWBI; Dyrbye et al., 2013) developed by the Mayo Clinic. The original measure includes seven dichotomous items designed to capture common indicators of strain, such as emotional detachment, depressive symptoms, and anxiety. For the purposes of this study, the PWBI’s initial burnout item (“Have you felt burned out from your work?”) was replaced with the single-item burnout measure from the Mini Z (Rohland et al., 2004). This item is scored on a five-point scale ranging from 1 (“I enjoy my work. I have no symptoms of burnout”) to 5 (“I feel completely burned out and may need to seek help”). Responses of 3 or higher were coded as indicating moderate to severe burnout and therefore contributed one point to the overall strain score. No further changes were made to the PWBI items beyond the replacement of the initial burnout item scores for mental health strain, which were averaged across the three monthly assessments to create a composite indicator of overall mental health strain.

National Emergency Department Overcrowding Scores (NEDOCS) were used to assess objective crowding levels during each month of the pandemic. The NEDOCS system is a weighted linear composite that factors in several variables, including the number of emergency department beds, the total number of hospital beds, the number of patients in the emergency department, the number of patients on mechanical ventilation, the length of stay of the patient with the longest visit, and the time since the last patient admission (Weiss et al., 2004). Ahalt et al. (2018) demonstrated NEDOCS’s effectiveness using a discrete-event simulation of a large academic Emergency Department (ED) in the southeastern United States. By comparing the simulation results with actual ED observations, they showed that NEDOCS accurately captures current crowding and can predict impending overcrowding. Scores range from 0, indicating “not busy,” 100, indicating “over-crowded,” to 200, representing “dangerously overcrowded” (Weiss et al., 2004).

## 3. Results

In January 2022, the Emergency Departments of the academic health center averaged a NEDOCS of 101.83, indicating overcrowdedness (Weiss et al., 2004). Two of the center’s hospital campuses, which represent half of our samples, reported a higher average of 153.94, indicating severe overcrowdedness. By February 2022, the surge started its decline with an average NEDOCS of 54.01 (indicating Busy), though the largest hospital campus still reported a high score of 126.18 (i.e., over-crowded). The reported NEDOCS scores objectively reflect the higher crowding levels associated with the Omicron surge. Table 2 provides the correlations between the measured variables at the two time periods. In line with expectations, perceived resilience was positively associated with meaningful work at Time 1, and both perceived resilience and meaningful work were negatively associated with mental health strain at Time 2.

To evaluate the relationship between pre-surge perceptions of resilience, meaningful work, and post-surge mental health strain, we conducted a hierarchical linear regression analysis controlling for gender, professional role, and the month in which participants first completed the resilience measure (see Table 3). A three-step hierarchical approach was utilized. Gender, professional role, and month of BRS completion were included in Step 1 to account for potential confounding factors that might influence the relationship between resilience, meaningful work, and mental health strain. Step 1 was not significant (*p* > 0.05). In Step 2, perceived resilience was added to the model. This model significantly predicted mental health strain, (R^2^ = 0.13, Adj. R^2^ = 0.10, *F*(7, 189) = 4.11, *p* < 0.001). In support of hypothesis 1, perceived resilience was a significant negative predictor of mental health strain (β = −0.320, *p* < 0.001). In Step 3, meaningful work was added to test whether it accounted for additional variance in mental health strain beyond resilience and control variables. The overall model continued to predict mental health strain significantly (*R*^2^ = 0.26, Adj. R^2^ = 0.23, *F*(8, 188) = 8.10, *p* < 0.001) with both resilience (β = −0.20, *p* = 0.004) and meaningful work (β = −0.40, *p* < 0.001) significantly predicting mental health strain. This supports Hypothesis 2; meaningful work was a significant predictor of lower mental health strain. No control variables were significant in any step. Because the addition of meaningful work reduced the effect of resilience, a formal mediation analysis was conducted to examine whether meaningful work mediates the relationship between resilience and mental health strain.

**Figure 2:**
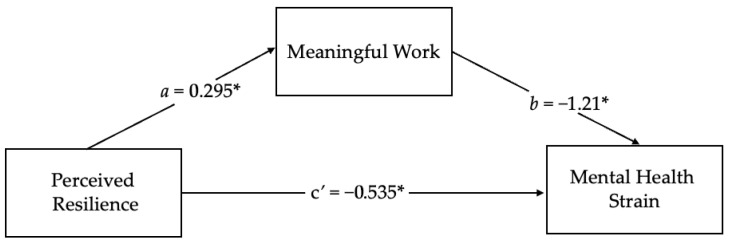
Visualization of the mediation results. Note. * *p* < 0.05.

A mediation analysis was conducted using PROCESS Macro Model 4 (Hayes, 2022) to examine whether meaningful work mediated the relationship between resilience and mental health strain. As seen in Figure 2 of the mediation results, perceived resilience significantly predicted meaningful work, *b* = 0.29, *p* < 0.001, and meaningful work significantly predicted lower mental health strain, *b* = −1.21, *p* < 0.001. After accounting for meaningful work, resilience continued to have a significant direct effect on mental health strain, *b* = −0.53, *p* = 0.008, indicating partial mediation. There was a significant indirect effect of resilience on mental health strain through meaningful work with meaningful work accounting for 40% of the total relationship. In support of Hypothesis 3, meaningful work partially mediated the relationship between perceived resilience and mental health strain. EM clinicians who perceived themselves as resilient were also likely to find meaning in their work, and that sense of meaning was tied to lower mental health strain.

## 4. Discussion

This study adds to the growing literature on psychological resilience in healthcare by demonstrating that EM clinicians who reported higher levels of perceived resilience prior to the Omicron COVID-19 surge experienced lower mental health strain in the subsequent months, despite the sustained and unpredictable nature of the stressors associated with the pandemic. The findings support the idea that resilience may shape how individuals respond to stressors. In line with appraisal-based perspectives, individuals with higher perceptions of pre-surge resilience may have been more likely to interpret difficult events as manageable rather than overwhelming.

An important takeaway from our findings is that resilience may be more than “toughness” or endurance. The findings of the present study suggest that one mechanism for the benefits of perceived resilience could be the creation of purpose and meaning even in the midst of chaos and uncertainty. In emergency medicine, where clinicians often face impossible decisions and constant ambiguity, a resilient mindset may help clinicians find or interpret meaning in patient care, teamwork, or their sense of professional identity (Horowitz et al., 2003; Pavlish & Hunt, 2012). These sources of meaning appear to function as an anchor that helps keep clinicians grounded when their environment is unpredictable (e.g., during the Omicron surge). This perspective may be especially critical in healthcare, where clinicians cannot always control external pressures but may be able to shape how they interpret and connect with their work.

### Limitations and Implications

The present study includes some limitations. First, the mediation model was tested with two time points, which constrains our ability to draw firm conclusions about the temporal ordering of resilience, meaningful work, and mental health strain. Our theoretical model proposed that perceived resilience predicts a greater tendency to experience meaningful work under high levels of adversity, but the fact that the two constructs were assessed at the same time prevented a thorough test of this directionality. Although our longitudinal design reduces concerns about reverse causality, a more rigorous test of mediation with at least three time points is suggested. Future studies should incorporate additional measurement waves to fully understand how resilience contributes to the development of meaningful work and how meaningful work predicts changes in mental health outcomes. Second, the utilization of self-report measures may have introduced common method bias (Podsakoff et al., 2003). However, the temporal separation between the predictors and outcome helps mitigate this concern (Podsakoff et al., 2003). Another limitation is that this study used a meaningful work scale developed specifically for this research. The scale demonstrated a clear factor structure and good internal consistency reliability. However, future research should aim to utilize or develop a validated, meaningful work scale for healthcare workers to strengthen measurement rigor. Additionally, a major limitation of the study is that 86 percent of participants identified as White. This percentage is reflective of the broader demographics of the geographic area where data were collected, but it limits the generalizability of the findings. Future research should aim to recruit a more diverse sample to strengthen the applicability of results. Finally, our sample was drawn from emergency clinicians during a highly specific crisis context. This may limit generalizability to other healthcare settings or circumstances. Future research should explore resilience, meaningful work, and mental health strain across a variety of healthcare settings and crisis situations.

Despite these limitations, results from this study provide important implications for further research and practice. Our results suggest that resilience initiatives may be stronger if paired with strategies that enhance perceptions of meaning in work. Reflection sessions, storytelling, or opportunities for peer recognition could help clinicians reconnect with the deeper purpose of their roles, especially during high-stress periods (Drutchas et al., 2024; Hollinger-Smith et al., 2021; Kennett & Lomas, 2015; Montani et al., 2017).

It is also important to recognize that focusing solely on individual resilience places responsibility on healthcare workers to manage their own mental health. Organizational-level interventions are equally critical (Munn et al., 2021; Panagioti et al., 2017). Evidence suggests that interventions targeting the healthcare system through means such as structural changes, fostering communication, enhancing teamwork, and promoting job control are more effective in reducing burnout than individual-directed approaches alone (Panagioti et al., 2017). Healthcare leaders should aim to reduce excessive demands, provide adequate resources, and foster supportive work environments to further equip employees to maintain well-being while navigating high-pressure situations such as a pandemic or extreme patient surge contexts. By addressing both personal and systemic factors, interventions can more effectively promote clinician well-being.

Finally, it is worth noting that the relationship between resilience, meaning, and strain is likely cyclical. The present study supports the idea that resilience helps foster meaningful work, while Arslan and Yıldırım (2021) suggest the reverse direction, showing that experiencing a meaningful life can also enhance resilience over time. Future research should track these processes across multiple stress cycles (e.g., flu surges, mass casualty events) to see how they evolve and whether interventions can reinforce both resilience and meaningfulness in a way that creates long-term benefits.

## 5. Conclusions

Overall, this study highlights the role of employee perceptions of resilience and meaningful work in predicting mental health strain among healthcare workers during the COVID-19 Omicron surge. Findings provide additional evidence for the predictive validity of the BRS. The results also suggest that a perception of work as meaningful is one of the factors accounting for the beneficial effects of perceived resilience. These findings support the potential value of interventions that enhance resilience by building a sense of meaningful work. Future research should seek to examine the effectiveness of such interventions designed to enhance the perceived resilience of healthcare providers by elevating the meaningfulness of their work.

## Figures and Tables

**Table 1:** Demographic characteristics of the participants.

Sample Characteristics	*n*	*%*	*M*	*SD*
Gender				
Male	129	65.5%		
Female	68	34.5%		
Professional Role				
Advanced Practice Clinicians	18	9.1%		
Attending	71	36%		
Resident	14	7.1%		
Registered Nurses	94	47.7%		
Race				
Asian	7	3.6%		
Black or African American	7	3.6%		
Hispanic or Latino	3	1.5%		
Two or More Races	10	5.1%		
White	170	86.3%		
Age			38.65	11.48

Note. *n* = 197.

**Table 2:** Descriptive statistics and intercorrelations for relevant study variables.

Variables	*M*	*SD*	1		2		3
Time 1							
1. Brief Resilience	3.60	0.60	-				
2. Meaningful Work	3.65	0.61	0.29	***	-		
Time 2							
3. Mental Health Strain	2.32	1.84	−0.29	***	−0.46	***	-

Note. *** *p* < 0.001.

**Table 3:** Results of hierarchical linear regression analysis predicting mental health strain.

Predictor	*b*	SE *b*	β	*t*	*p*
Step 1					
Constant	5.76	0.77	-	7.49	<0.001
Role: APC	0.07	0.45	0.01	0.16	0.87
Role: Attending	−0.42	0.30	−0.11	−1.41	0.16
Role: Resident	−0.91	0.52	−0.13	−1.75	0.08
Gender: Male	0.47	0.29	0.12	1.63	0.10
Month: January	−0.30	0.64	−0.03	−0.47	0.64
Month: December	0.59	0.32	0.13	1.86	0.06
Step 2					
Perceived Resilience	−0.97	0.21	−0.32	−4.66	<0.001
Step 3					
Perceived Resilience	−0.60	0.21	−0.20	−2.92	0.004
Meaningful Work	−1.15	0.21	−0.38	−5.60	<0.001

Note. *b* = unstandardized regression coefficient; SE = standard error; β = standardized regression coefficient.

## Data Availability

The raw data supporting the conclusions of this article will be made available by the authors upon request to protect privacy, as open-ended comments could potentially identify individuals or hospitals.
